# Relationship Between Polypharmacy and Quality of Life Among People in 24 Countries Living With HIV

**DOI:** 10.5888/pcd17.190359

**Published:** 2020-03-05

**Authors:** Chinyere Okoli, Patricia de los Rios, Anton Eremin, Gary Brough, Benjamin Young, Duncan Short

**Affiliations:** 1 ViiV Healthcare, Brentford Middlesex, United Kingdom; 2 ViiV Healthcare, Research Triangle Park, North Carolina; 3 AIDS Center Foundation, Moscow, Russia; 4 UK CAB and Positively UK, London, United Kingdom

## Abstract

**Introduction:**

People living with HIV (PLHIV) have greater risk of having multiple health conditions. We measured the relationship between increased medication and overall quality of life among PLHIV from 24 countries.

**Methods:**

We analyzed data for 2,112 adult PLHIV on antiretroviral therapy (ART) in 24 countries who completed the 2019 Positive Perspectives survey. Polypharmacy was defined as taking 5 or more pills a day or currently taking medications for 5 or more conditions. Outcomes were self-rated overall health, treatment satisfaction, and self-reported virologic control. New treatment concerns were issues not prioritized at ART initiation but now deemed paramount. Data were analyzed with descriptive and multivariable statistics.

**Results:**

Overall prevalence of polypharmacy was 42.1%. People reporting polypharmacy had significantly poorer health outcomes independent of existing comorbidities; their odds of treatment satisfaction, optimal overall health, and virologic control were lower by 27.0% (adjusted odds ratio [AOR] = 0.73; 95% CI, 0.59–0.91), 36.0% (AOR = 0.64; 95% CI, 0.53–0.78), and 46.0% (AOR = 0.54, 95% CI, 0.42–0.70), respectively, compared with those without polypharmacy (all *P* < .05). Most PLHIV (56.6%) were concerned about taking more medicines as they age, and 73.1% were interested in ARTs with fewer medicines. Top reasons for switching ART among those who had ever switched (n = 1,550) were to reduce severity and frequency of side effects (45.3%), number of pills (35.0%), or number of medicines (26.8%). People reporting polypharmacy had significantly higher odds of having new concerns relative to when they initiated ART, regarding risks of drug–drug interactions (AOR = 1.32; 95% CI, 1.02–1.71) and side effects (AOR = 1.31; 95% CI, 1.02–1.68).

**Conclusion:**

Polypharmacy was associated with poorer health-related outcomes among PLHIV. Many PLHIV expressed concerns about side effects of ART. Clinicians should carefully consider patient preferences, comorbidities, and drug profiles when prescribing ART.

**Summary**

**What is already known on this topic?**

People living with HIV (PLHIV) have greater incidence of comorbidities and higher prevalence of polypharmacy, most commonly defined as taking 5 or more medications concurrently, than the general population.

**What is added by this study?**

PLHIV reporting polypharmacy had significantly worse overall health outcomes, including significantly lower prevalence of self-reported virologic control and treatment satisfaction. These findings were significant despite controlling for presence of comorbidities.

**What are the implications for public health practice?**

By actively assessing patients holistically and considering how increased medication may influence individual behavior to health outcomes, clinicians may contribute to improving health-related quality of life among people living with HIV.

## Introduction

With advances in antiretroviral therapy (ART), the life expectancy of people living with HIV (PLHIV) has increased (1–4); nevertheless, they are at higher risk for multiple health disorders (5,6). As with many other chronic illnesses, HIV management goes beyond drug treatment; holistic care is paramount. In its definition of health, the World Health Organization shifted its focus from diagnosis-focused approaches to ones that highlight the person’s positive overall well-being and quality of life across physical, mental, and social domains (7). This paradigm has also emerged within the HIV community with the incorporation of the “fourth 90,” calling for improvements in health-related quality of life. The fourth 90 evolved from the global 90–90–90 targets of the Joint United Nations Programme on HIV/AIDS, which called on health systems to increase diagnosis of HIV, treat a greater number of those diagnosed, and ensure that those being treated achieve viral suppression. (8).

Focusing entirely on a specific disease reduces the health care provider’s ability to personalize care for a person affected by a host of comorbidities. Other than medical care, a person’s health is significantly determined by environmental, sociopsychological, and economic factors (9). Hence, success measured purely by clinical outcomes may not mitigate disease. Delivery of fragmented care to patients affected by multiple conditions, even when based on strict adherence to disease-oriented treatment guidelines, promotes polypharmacy, which may increase likelihood of adverse or even antagonistic treatment effects (10).

Because PLHIV have more non-AIDS comorbidities, a comprehensive approach is needed to evaluate the relationship between increased medication and overall quality of life. Polypharmacy rates among PLHIV, which range from 23% to 39%, are higher than among HIV-negative populations (11–15). Studies have demonstrated associations between polypharmacy and clinical outcomes, including death (16); however, data are scarce on the relationship between polypharmacy and overall quality of life based on subjective measures of well-being that are important to patients (17, 18), including their experiences, concerns, and treatment priorities. To fill these gaps in knowledge, we used pooled data from 24 countries to examine associations between polypharmacy and self-rated overall health and perceptions of treatment needs.

## Methods

### Data source

We conducted a cross-sectional study, Positive Perspectives, from April through August 2019 in 24 countries; 2,112 PLHIV aged 18 to 84 participated. Participating countries and the achieved sample size were United States (n = 400), United Kingdom (n = 123), Australia (n = 120), Canada (n = 120), France (n = 120), Germany (n = 120), Italy (n = 120), Spain (n = 120), Japan (n = 75), Mexico (n = 63), Portugal (n = 60), Brazil (n = 58), Russia (n = 57), Taiwan (n = 55), Netherlands (n = 51), Argentina (n = 50), Austria (n = 50), Chile (n = 50), China (n = 50), Ireland (n = 50), Belgium (n = 50), Poland (n = 50), South Korea (n = 50), and Switzerland (n = 50). To be eligible, respondents had to verify that they were HIV sero-positive and receiving treatment (eg, by presenting their ART prescription or a letter from their medical provider); those not currently on HIV treatment (31.6%) were excluded. Financial incentives (approximately £20 GBP or the local equivalent) were provided to encourage participation. Participants were recruited by using targeted and snowball sampling approaches across multiple platforms and in collaboration with multiple HIV organizations; responses were collected over the web or in person. Adaptive sampling was performed to make the distributions of participants in each country more representative. This study was deemed exempt research by the Pearl Institutional Review Board (no. 18–080622).

### Measures

**Polypharmacy**. Polypharmacy has no universal definition (19); however, one commonly used definition is 5 or more medications concurrently (20). We defined polypharmacy as taking 5 or more pills a day or taking medicines for 5 or more health conditions. This definition, which distinguishes pills (dosage form of a medicine) from medicines (the actual pharmacologic agent) is conservative inasmuch as a single pill could contain more than 1 medicine, which is likely with ART (21), and more than 1 medication could be used to treat a single condition. Survey participants were asked their frequency of taking HIV and non-HIV pills and were also shown a detailed list of health conditions to ascertain which ones they were currently taking medicines for. Polypharmacy was assessed independent of the presence of diagnosed comorbidities; for example, people taking 5 or more medications for 1 condition would be classified as reporting polypharmacy, whereas those with 5 or more conditions but taking fewer than 5 medicines would be classified as not reporting polypharmacy.

We created 2 other proxies of polypharmacy: a cognitive marker (perceived overmedication), and a behavioral marker (cut-down attempt). The cognitive proxy asked 6 questions to assess respondents’ anxiety regarding 1) short-, medium-, and long-term effects of their current HIV medicines, including long-term side effects; 2) prospect of having to take increasingly more medicines; 3) potential interactions among medications; 4) potential effect on body or body shape; 5) potential effect on overall health and well-being; and 6) unknown long-term effects. People were classified as having a perception of overmedication if they affirmed on at least 5 of these questions that they were worried about their intake of medications. 

The behavioral proxy (cut-down attempt) was based on the premise that skipping or completely switching medication for the sole purpose of reducing intake of medicines could indicate a person’s real or perceived awareness that their medicine intake was high. People were classified as having made a cut-down attempt if they reported skipping their HIV medicines in the past 30 days because they were concerned about medication side effects or if they completely switched regimens to reduce the number of medicines taken.

**Self-rated overall health and its composite domains**. Participants were asked “How would you describe your [physical/mental/sexual/overall] health over the past 4 weeks?” Response options were the same for physical, mental, sexual, and overall health: very poor, poor, neither good nor poor, good, or very good. Self-ratings of good or very good were classified as optimal health on that domain; all other responses were classified as suboptimal.

**Treatment concerns, satisfaction, and aspirations**. PLHIV were classified as being recent initiates, treatment experienced, or very treatment experienced. Recent initiates were PLHIV who had been diagnosed within the past 2 years and were on their first regimen. Very treatment experienced were people who switched ART 4 or more times and stopped the penultimate regimen after less than 1 year because of issues with frequency or severity of side effects, noneffectiveness, or resistance. PLHIV not meeting the definition of very treatment experienced or recent initiates were classified as treatment experienced. The survey further assessed participants’ awareness of the number of HIV medicines contained in single pills or medications.

“Treatment satisfaction” and “met treatment needs” were defined with affirmative responses to the following questions: “Overall, how satisfied are you with your current HIV medication?” “When it comes to the management of your HIV treatment, do you feel your main HIV care provider meets your personal needs and takes into account the things that are most important to you?” A pessimistic outlook was an affirmative response to at least one of the following: “HIV will reduce my life span,” or “Because of my HIV, I do not plan for my old age.”

The survey further asked participants about what they prioritized the most when they started ART and what their current priorities were. A new treatment concern was an issue that was not deemed important by the respondent at ART initiation but was currently considered a treatment priority.

**Self-reported virologic status**. Self-reported virologic control was defined as a response of “undetectable,” “suppressed versus detectable,” “unsuppressed,” “I don’t know,” or “prefer not to say” to the question “What is your most recent viral load?”

**Demographic characteristics**. Demographic characteristics were age, gender, sexual orientation, geographic region, urbanicity, race, employment status, year of diagnosis, disease duration, and non-HIV comorbidities.

### Statistical analyses

Descriptive and multivariable analyses were performed to measure the relationships between polypharmacy and measures of overall well-being; subgroup differences were tested with χ^2^ tests. Multivariable logistic regression analyses adjusted for age, duration of disease, geographic region, comorbidities, urbanicity, education, and sexual orientation. We performed stratified analyses among PLHIV reporting virologic control (n = 1,536) and those not reporting virologic control (n = 576). Significance was set at *P* < .05. All analyses were performed with R Version 3.1.1 (The R Foundation).

## Results

A total of 2,112 people completed our survey (Table 1). Overall, 70.4% were male, 61.4% were white, 45.1% were men who have sex with men, 30.7% were aged 50 or older, and 23.1% were diagnosed with HIV in the past 2 years. Geographically, 45.6% of the sample were from the European Union, 27.6% were from North America, and 26.8% were from other parts of the world. The percentage reporting optimal health was as follows by the health domain: physical, 58.1%; mental, 56.1%; sexual, 46.8%; and overall, 55.8%; approximately 58.9% had less than 1 non-HIV comorbidity. The most common non-HIV conditions PLHIV reported ever being diagnosed with were mental health disorders, including anxiety and depression (20.6%); hypertension (17.3%); hypercholesterolemia (16.9%); insomnia or other sleep disorders (15.6%); gastrointestinal diseases, including ulcers, reflux, and Crohn’s disease (12.5%); anemia (11.5%); liver disease (10.8%); arthritis (9.4%); and lung/respiratory diseases (9.1%). 

**Table 1:** Sociodemographic and Clinical Characteristics of People Living With HIV (N = 2,112) in 24 Countries and Measures of Self-Rated Health, Positive Perspectives Study, 2019^a^

Variable	Subjective Measures of Quality of Life, %					
	No.	Optimal Overall Health^b^	Optimal Mental Health^b^	Optimal Sexual Health^b^	Optimal Physical Health^b^	Treatment Satisfaction
**Total**	2,112	55.8	56.1	46.8	58.1	69.6
**Gender**						
Male	1,486	58.1	56.1	46.6	60.1	72.5
Female	571	50.1	54.6	46.4	51.1	62.9
Other	55	54.5	70.9	58.2	78.2	60.0
**Sexual orientation**						
Heterosexual	804	48.5	47.1	43.2	48.4	59.0
Homosexual	1,023	61.8	61.4	48.1	65.3	77.8
Other	285	55.1	62.5	52.6	60.0	70.2
**Gender/sexual orientation**						
Men who have sex with men	953	62.4	61.7	48.2	66.3	78.4
Men who have sex with women	420	50.7	43.8	44.8	48.8	60.0
Women who have sex with women	58	45.8	50.1	41.5	47.2	57.7
Women who have sex with men	369	51.7	56.9	43.1	48.3	69.0
Other/indeterminate	312	55.1	62.5	52.6	60.6	69.9
**Age, y**						
<50	1,464	57.2	54.1	50.6	59.7	67.6
≥50	648	52.8	60.6	38.3	54.6	74.1
**Year of HIV diagnosis**						
2017–2019	488	51.8	53.1	46.9	56.4	64.1
2010–2016	805	58.4	55.7	51.6	58.6	70.3
Before 2010	819	55.7	58.4	42.1	58.7	72.2
**Geographic region**						
European Union	964	57.5	60.3	51.2	62.8	72.1
North America	583	55.6	50.1	39.5	52.5	71.2
Other	565	53.3	55.2	46.9	56.1	63.7
**Home ownership**						
Own	653	55.7	56.7	44.7	55.4	72.0
Rent	825	57.8	57.3	47.9	61.3	70.9
Other	634	53.3	53.9	47.6	56.8	65.5
**Urbanicity**						
Metropolitan	1,170	57.1	59.4	48.1	61.4	73.0
Nonmetropolitan	942	54.2	52.0	45.2	54.1	65.4
**Employment status**						
Employed	1,258	61.1	58.5	51.2	62.2	72.6
Unemployed/not in workforce	854	48.0	52.6	40.4	52.1	65.2
**Race**						
Nonwhite	816	51.7	54.2	44.6	57.6	63.4
White	1296	58.4	57.3	48.2	58.5	73.5
**Comorbidity, no.**						
None	879	64.6	58.9	57.1	64.8	72.0
1	464	59.1	59.5	48.9	65.3	70.9
≥2	769	43.8	50.8	33.8	46.2	66.1
**Comorbidity, type**						
None	879	64.6	58.9	57.1	64.8	72.0
Cardio-hematologic only	177	62.1	69.5	53.7	67.8	70.1
Bone only	23	52.2	69.6	47.8	52.2	56.5
Neurologic only	151	53.6	44.4	43.0	65.6	72.2
Gastrointestinal only	56	55.8	63.5	44.2	57.7	73.1
Pulmonary only	23	73.9	60.9	69.6	78.3	78.3
Cancer only	15	73.3	66.7	53.3	73.3	80.0
Endocrine/renal only	23	60.9	56.5	39.1	56.5	65.2
Multiple	765	43.8	50.8	33.8	46.2	66.1
**Concomitant medications**						
None	1,209	63.4	59.1	53.8	64.6	71.5
1	419	55.8	58.0	47.7	60.1	67.1
≥2	484	37.0	46.9	28.7	40.3	66.9
**Antiretroviral therapy, switch history, no. of times**						
None	562	59.6	64.8	55.2	66.0	67.6
1	563	60.4	51.5	50.8	60.6	69.4
2	390	49.7	45.6	42.8	46.4	66.7
3	235	57.4	57.0	44.3	61.3	78.7
≥4	362	48.3	60.5	33.7	52.8	70.2
**Antiretroviral therapy, experience** c						
Recent initiate	241	61.0	67.6	54.4	66	69.7
Experienced	1,790	55.4	54.2	46.1	57.3	70.0
Very experienced	81	50.6	63.0	40.7	53.1	60.5
**Self-reported viral load**						
Unsuppressed/unknown	576	49.0	47.6	42.5	46.9	63.7
Suppressed	1,536	58.4	59.3	48.4	62.4	71.8

a Participating countries were Argentina, Austria, Australia, Belgium, Brazil, Canada, Chile, China, France, Germany, Ireland, Italy, Japan, Mexico, Netherlands, Poland, Portugal, Russia, South Korea, Spain, Switzerland, Taiwan, United Kingdom, and United States.

b Self-rated health as good or very good was classified as optimal; ratings of very poor, poor, or neither good nor poor were classified as suboptimal health.

c Recent initiates were people living with HIV (PLHIV) who had been diagnosed within the past 2 years and were on their first regimen. Very treatment experienced were people who switched antiretroviral therapy 4 or more times and stopped the penultimate regimen after less than 1 year because of issues with frequency or severity of side effects, noneffectiveness, or resistance. PLHIV not meeting the definition of very treatment experienced or recent initiates were classified as treatment experienced.

Of all participants, 82.0% reported taking at least one non-HIV pill per day or taking medication currently for at least one non-HIV condition, in addition to their ART. The mean number of pills (HIV and non-HIV combined) per day reported was 4.25 (standard deviation [SD], 2.28). By polypharmacy status, average pill count per day was 6.48 (95% confidence interval [CI], 6.39–6.57) among people with a report of polypharmacy and 2.63 (95% CI, 2.57–2.69) among those without. Overall prevalence of polypharmacy was 42.1%, and this was highest among the following subgroups: men who have sex with women (50.5%, 211 of 418) compared with men who have sex with men (38.9%, 369 of 949) and women (42.1%, 239 of 568) (*P* < .001); people aged 50 or older (54.6%, 352 of 645) compared with those younger than 50 (36.5%, 532 of 1,457) (*P* < .001); people diagnosed with HIV before 2010 (50.3%) compared with those diagnosed between 2017 and 2019 (33.5%, 163 of 487) or between 2010 and 2016 (38.9%, 312 of 802) (*P* < .001); people living in North America (52.2%, 304 of 582) compared with those living in the European Union (33.6%, 323 of 960) or other regions (45.9%, 257 of 560) (*P* < .001); people residing in nonmetropolitan areas (45.2%, 424 of 938) compared with metropolitan areas (39.5%, 460 of 1,164) (*P* = .009); and people unemployed or not in the work force (48.1%, 409 of 850) compared with those employed (37.9%, 475 of 1,252) (*P* < .001) (Table 2). Furthermore, prevalence of polypharmacy was 26.8%, 36.8%, and 62.8% among those with 0, 1, or 2 or more non-HIV comorbidities, respectively (*P* < .001). Results from the 2 proxy definitions for polypharmacy yielded results very similar to those obtained with the more objective count of medications; prevalence was 45.7% with the cognitive proxy (perceived overmedication) and 43.7% with the behavioral proxy (cut-down attempt).

**Table 2:** Polypharmacy and Perceptions of HIV Medications Among People Living With HIV (N = 2,112) in 24 Countries, Positive Perspectives Study, 2019^a^

Variable	Polypharmacy and Concerns About Medicines, %						
	No.	Polypharmacy^ b^	Awareness of No. of Medicines in Their Daily Regimen	Worry About More Medicines	Worry About Long-Term Effects of Medicines	Worry About Drug–Drug Interactions	Open to Taking Fewer HIV Medicines
**Total**	2,112	42.1	75.1	56.6	66.6	48.5	73.1
**Gender**							
Male	1,486	42.8	77.0	55.7	66.2	46.5	73.9
Female	571	42.1	72.7	58.7	67.4	52.5	71.5
Other	55	20.4	49.1	60.0	69.1	60.0	69.1
**Sexual orientation**							
Heterosexual	804	48.3	71.0	55.3	66.2	49.1	67.5
Homosexual	1,023	38.7	79.9	56.4	67.4	45.7	76.9
Other	285	36.5	69.5	60.7	64.9	56.5	75.1
**Gender/sexual orientation**							
Men who have sex with men	953	38.9	80.4	56.2	68.3	45.2	77.9
Men who have sex with women	420	50.5	68.3	53.8	61.0	47.9	63.8
Women who have sex with women	58	47.0	74.0	56.6	71.5	49.9	71.3
Women who have sex with men	369	37.9	77.6	58.6	55.2	51.7	63.8
Other/indeterminate	312	35.4	68.9	60.9	65.1	57.1	75.0
**Age, y**							
<50	1,464	36.5	72.3	57.7	65.9	50.8	70.1
≥50	648	54.6	81.3	54.2	68.1	43.4	79.9
**Year of HIV diagnosis**							
2017–2019	488	33.5	75.4	58.8	63.7	53.3	72.5
2010–2016	805	38.9	73.4	56.3	64.1	49.9	70.1
Before 2010	819	50.3	76.6	55.6	70.7	44.2	76.4
**Geographic region**							
European Union	964	33.6	70.5	55.5	65.5	43.8	73.9
North America	583	52.2	80.4	55.6	62.8	49.1	69.8
Other	565	45.9	77.3	59.5	72.4	55.9	75.2
**Home ownership**							
Own	653	50.5	81.8	55.1	66.9	47.2	75.7
Rent	825	38.9	73.8	55.6	66.1	47.6	75.8
Other	634	37.4	69.9	59.3	66.9	50.9	67.0
**Urbanicity**							
Metropolitan	1,170	39.5	73.8	59.0	71.2	49.4	76.3
Nonmetropolitan	942	45.2	76.6	53.6	60.8	47.3	69.1
**Employment status**							
Employed	1,258	37.9	76.1	54.5	63.8	47.5	74.5
Unemployed/not in workforce	854	48.1	73.7	59.7	70.7	50.0	71.1
**Race**							
Nonwhite	816	38.9	70.3	60.7	73.2	52.1	70.0
White	1296	44.0	78.1	54.0	62.4	46.2	75.1
**Comorbidity, no.**							
None	879	26.8	70.3	52.7	58	45.1	68.8
1	464	36.8	73.5	55.4	68.5	47.6	73.3
≥2	769	62.8	81.5	61.8	75.2	52.9	77.9
**Comorbidity, type**							
None	879	26.8	70.3	52.7	58.0	45.1	68.8
Cardio-hematologic only	177	39.2	68.4	56.5	68.9	52.0	69.5
Bone only	23	34.8	91.3	34.8	65.2	34.8	82.6
Neurologic only	151	36.4	72.8	62.9	72.8	49.7	73.5
Gastrointestinal only	56	25.5	78.8	46.2	61.5	40.4	82.7
Pulmonary only	23	43.5	82.6	52.2	69.6	47.8	65.2
Cancer only	15	20.0	73.3	40.0	60.0	40.0	80.0
Endocrine/renal only	23	52.2	78.3	52.2	60.9	34.8	73.9
Multiple	765	62.8	81.5	61.8	75.2	52.9	77.9
**Concomitant medications**							
None	1,209	27.6	71.6	52.7	61.5	45.9	70.3
1	419	42.0	75.1	58.7	70.9	48.0	77.8
≥2	484	78.5	84.6	64.1	74.9	54.9	76.0
**Switched antiretroviral therapy, no. of times **							
None	562	19.9	62.8	55.0	62.1	46.1	67.4
1	563	43.7	75.7	52.4	62.7	48.7	67.0
2	390	49.4	77.9	60.5	66.2	54.9	75.4
3	235	44.7	79.6	57.4	71.5	45.5	80.4
≥4	362	64.4	87.3	60.8	76.8	47.0	84.3
**Antiretroviral therapy, experience with**							
Recent initiates	241	19.1	73.4	52.3	59.3	45.6	75.5
Experienced	1,790	44.5	74.7	56.7	66.9	48.9	72.1
Very experienced	81	56.2	88.9	66.7	80.2	46.9	88.9
**Self-reported viral load**							
Unsuppressed/unknown	576	46.8	76.6	54.7	56.2	51.6	71.0
Suppressed	1,536	40.3	74.5	57.3	70.4	47.3	73.9

a Participating country were Argentina, Austria, Australia, Belgium, Brazil, Canada, Chile, China, France, Germany, Ireland, Italy, Japan, Mexico, Netherlands, Poland, Portugal, Russia, South Korea, Spain, Switzerland, Taiwan, United Kingdom, and United States.

b Polypharmacy was defined as taking 5 or more pills per day for HIV or non-HIV conditions, or taking medicines for 5 or more conditions, including HIV.

**Figure 1:**
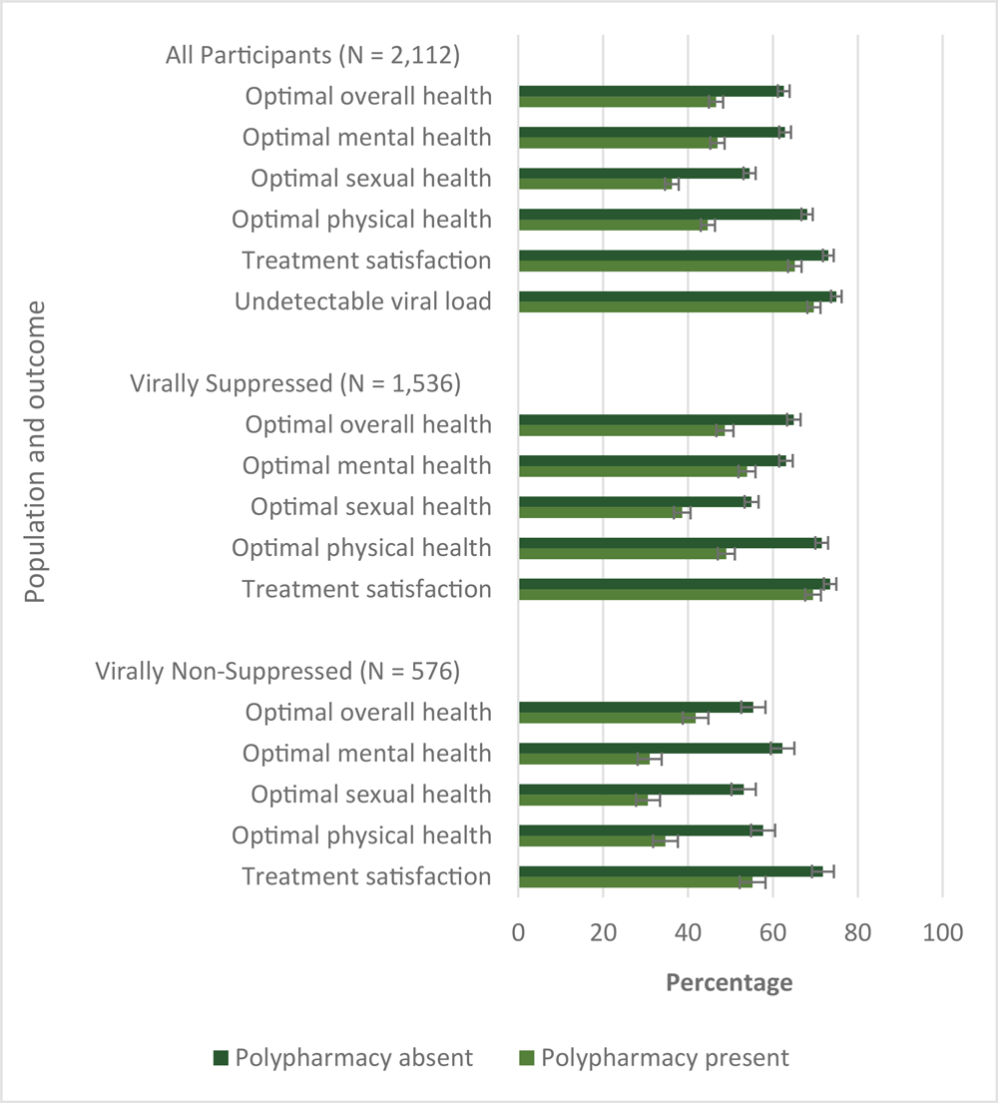
Comparison of prevalence of subjective measures of overall well-being by polypharmacy status, people from 24 countries who were living with HIV (N = 2,112), Positive Perspectives survey. Polypharmacy was defined as taking 5 or more pills per day for HIV or non-HIV conditions, or taking medicines currently for 5 or more conditions, including HIV. All differences between people with a report of polypharmacy compared with those not reporting polypharmacy were significant at *P* < .05. Brackets indicate standard errors.

**Polypharmacy and its association with measures of overall well-being**. PLHIV reporting polypharmacy had worse measures of overall well-being than those not reporting polypharmacy (Table 3). Prevalence estimates were as follows for those reporting compared with those not reporting polypharmacy, respectively: optimal overall health (46.6% vs 62.6%, *P* < .001), optimal mental health (46.9% vs 62.9%, *P* < .001), optimal sexual health (36.2% vs 54.5%, *P* < .001), and optimal physical health (44.7% vs 68.1%, *P* < .001). PLHIV reporting polypharmacy also had lower probability of reporting treatment satisfaction (65.2% vs 73.1%, *P* < .001), positive outlook (42.5% vs 49.8%, *P* = .001), and virologic control (69.7% vs 75.0%, *P* = .007). Consistent results were seen when analyses were stratified by self-reported virologic status (Table 3) (Figure 1).

**Table 3:** Prevalence of Study Outcomes Among People Living With HIV in 24 Countries^a^, With or Without a Report of Polypharmacy^b^, Stratified by Self-Reported Virologic Control Status^c^, Positive Perspectives Study, 2019

Outcome	Medication-Count–Based Measure of Polypharmacy			Proxy Measure of Polypharmacy	
	Prevalence of Outcome Among Those Reporting Polypharmacy (n = 884), %	Prevalence of Outcome Among Those Not Reporting Polypharmacy (n = 1,218), %	Associations With Polypharmacy and Outcome, AOR (95% CI)^d^	Associations Between Perceived Overmedication and Outcome, AOR (95% CI)^d^	Associations Between Cut-Down Attempt and Outcome, AOR (95% CI)^d^
**All participants (N = 2,112)**					
Self-rated overall health	46.6	62.6	0.64 (0.53–0.78)^e^	0.66 (0.55–0.80)^e^	0.66 (0.54–0.80)^e^
Self-rated mental health	46.9	62.9	0.58 (0.48–0.71)^e^	0.84 (0.70–1.01)	0.50 (0.42–0.61)^e^
Self-rated sexual health	36.2	54.5	0.63 (0.52–0.77)^e^	0.69 (0.58–0.83)^e^	0.57 (0.47–0.69)^e^
Self-rated physical health	44.7	68.1	0.49 (0.40–0.60)^e^	0.95 (0.79–1.14)	0.62 (0.51–0.76)^e^
Treatment satisfaction	65.2	73.1	0.73 (0.59–0.91)^e^	0.72 (0.59–0.87)^e^	0.57 (0.47–0.70)^e^
Met treatment needs	68.7	68.5	0.96 (0.78–1.18)	0.74 (0.61–0.89)^e^	0.68 (0.55–0.83)^e^
Positive outlook for longevity	42.5	49.8	0.85 (0.70–1.04)	0.38 (0.31–0.46)^e^	0.47 (0.39–0.57)^e^
**Reporting virologic suppression (n = 1,536)**					
Self-rated overall health	48.7	65.0	0.65 (0.51–0.83)^e^	0.61 (0.49–0.76)^e^	0.74 (0.59–0.93)^e^
Self-rated mental health	53.9	63.1	0.75 (0.59–0.95)^e^	0.72 (0.58–0.89)^e^	0.55 (0.44–0.68)^e^
Self-rated sexual health	38.6	55.0	0.75 (0.59–0.95)^e^	0.61 (0.49–0.75)^e^	0.60 (0.48–0.75)^e^
Self-rated physical health	49.0	71.5	0.53 (0.42–0.68)^e^	0.75 (0.60–0.93)^e^	0.68 (0.54–0.86)^e^
Treatment satisfaction	69.5	73.5	0.75 (0.58–0.98)^e^	0.67 (0.53–0.85)^e^	0.52 (0.41–0.66)^e^
Met treatment needs	72.2	68.1	1.05 (0.81–1.36)	0.65 (0.52–0.82)^e^	0.74 (0.58–0.94)^e^
Positive outlook for longevity	49.5	56.1	0.93 (0.74–1.18)	0.37 (0.30–0.45)^e^	0.56 (0.45–0.70)^e^
**Not reporting virologic suppression (n = 576)**					
Self-rated overall health	41.8	55.4	0.73 (0.50–1.07)	0.75 (0.52–1.09)	0.59 (0.40–0.87)^e^
Self-rated mental health	31.0	62.3	0.37 (0.25–0.54)^e^	1.35 (0.93–1.98)	0.48 (0.32–0.71)^e^
Self-rated sexual health	30.6	53.1	0.44 (0.29–0.66)^e^	0.88 (0.60–1.30)	0.54 (0.36–0.82)^e^
Self-rated physical health	34.7	57.7	0.48 (0.32–0.70)^e^	1.62 (1.11–2.36)^e^	0.61 (0.41–0.90)^e^
Treatment satisfaction	55.2	71.8	0.63 (0.43–0.94)^e^	0.92 (0.62–1.36)	0.73 (0.48–1.11)
Met treatment needs	60.4	69.5	0.70 (0.47–1.04)	0.99 (0.67–1.45)	0.51 (0.34–0.78)^e^
Positive outlook for longevity	26.5	30.8	0.94 (0.62–1.42)	0.26 (0.17–0.41)^e^	0.31 (0.21–0.48)^e^

Abbreviations: AOR, adjusted odds ratio; CI, confidence interval.

a Countries included in study were Argentina, Austria, Australia, Belgium, Brazil, Canada, Chile, China, France, Germany, Ireland, Italy, Japan, Mexico, Netherlands, Poland, Portugal, Russia, South Korea, Spain, Switzerland, Taiwan, United Kingdom, and United States.

b Polypharmacy was defined as taking 5 or more pills per day for HIV or non-HIV conditions or taking medicines currently for 5 or more conditions, including HIV/AIDS. For the cognitive proxy (perceived overmedication), we used a set of 6 questions that assessed respondents’ anxieties regarding short-, medium-, and long-term effects from their current HIV medicines, including worries about long-term side effects, having to take increasingly more medicines, potential interactions with other medications, impact on body and/or body shape, impact on overall health and well-being, and unknown long-term impact. Being worried about at least 5 of these items was subjectively classified as acute awareness on the part of the respondent of the number of medicines they were taking. For the behavioral proxy (cut-down attempt), participants were classified as having taken deliberate efforts to reduce the number/impact of medicines they were taking if they skipped taking their HIV medicines in the past 30 days because they were concerned about short- or long-term side effects of medicines or if they completely switched medicines to reduce the number of medicines taken.

c Self-reported virologic suppression was defined as a response of “undetectable” or “suppressed” versus “detectable,” “unsuppressed,” “I don’t know,” or “prefer not to say” to the question, “What is your most recent viral load?”

d Adjusted odds ratios for the different outcomes were computed in separate logistic regression models, controlling for age, duration of disease, geographic region, comorbidities, urbanicity, education, and sexual orientation.

e Significant at *P* < .05.

**Table:** 

Characteristics	Polypharmacy Absent, % (Standard Error)	Polypharmacy Present, % (Standard Error)
All participants (N = 2,112)		
Optimal overall health	62.6 (1.4)	46.6 (1.7)
Optimal mental health	62.9 (1.4)	46.9 (1.7)
Optimal sexual health	54.5 (1.4)	36.2 (1.6)
Optimal physical health	68.1 (1.3)	44.7 (1.7)
Treatment satisfaction	73.1 (1.3)	65.2 (1.6)
Undetectable viral load	75.0 (1.2)	69.7 (1.5)
Virally suppressed (n = 1,536)		
Optimal overall health	65.0 (1.6)	48.7 (2.0)
Optimal mental health	63.1 (1.6)	53.9 (2.0)
Optimal sexual health	55.0 (1.6)	38.6 (2.0)
Optimal physical health	71.5 (1.5)	49.0 (2.0)
Treatment satisfaction	73.5 (1.5)	69.5 (1.9)
Virally unsuppressed (n = 576)		
Optimal overall health	55.4 (2.9)	41.8 (3.0)
Optimal mental health	62.3 (2.8)	31.0 (2.8)
Optimal sexual health	53.1 (2.9)	30.6 (2.8)
Optimal physical health	57.7 (2.8)	34.7 (2.9)
Treatment satisfaction	71.8 (2.6)	55.2 (3.0)

After adjusting for potential confounders, including presence of comorbidities, people reporting polypharmacy had significantly worse overall health outcomes and lower odds of reporting virologic control and treatment satisfaction than those who did not. Those reporting polypharmacy compared with those not reporting it in the overall population had lower odds of self-rated optimal overall health (AOR = 0.64; 95% CI, 0.53–0.78), optimal mental health (AOR = 0.58; 95% CI, 0.48–0.71), optimal sexual health (AOR = 0.63; 95% CI, 0.52–0.77), and optimal physical health (AOR = 0.49; 95% CI, 0.41–0.60) (Table 3). Analyses of other measures of overall well-being revealed consistent results. People reporting polypharmacy had significantly lower odds, independent of presence of comorbidities, of treatment satisfaction (AOR = 0.73; 95% CI, 0.59–0.91) and self-reported virologic control (AOR = 0.54; 95% CI, 0.42–0.70). Stratified analyses on self-reported virologic control status showed that regardless of virologic control, people taking more medicines had less favorable health outcomes and treatment satisfaction (Figure 1). The association between polypharmacy and measures of overall well-being was, however, stronger among those not reporting than reporting virologic control. For example, polypharmacy reduced the odds of reporting optimal mental health by 25% (AOR = 0.75; 95% CI, 0.59–0.95) among those reporting virologic control, but by 63% among those reporting virologic failure (AOR = 0.37; 95% CI, 0.25–0.54) (Table 3).

**Figure 2:**
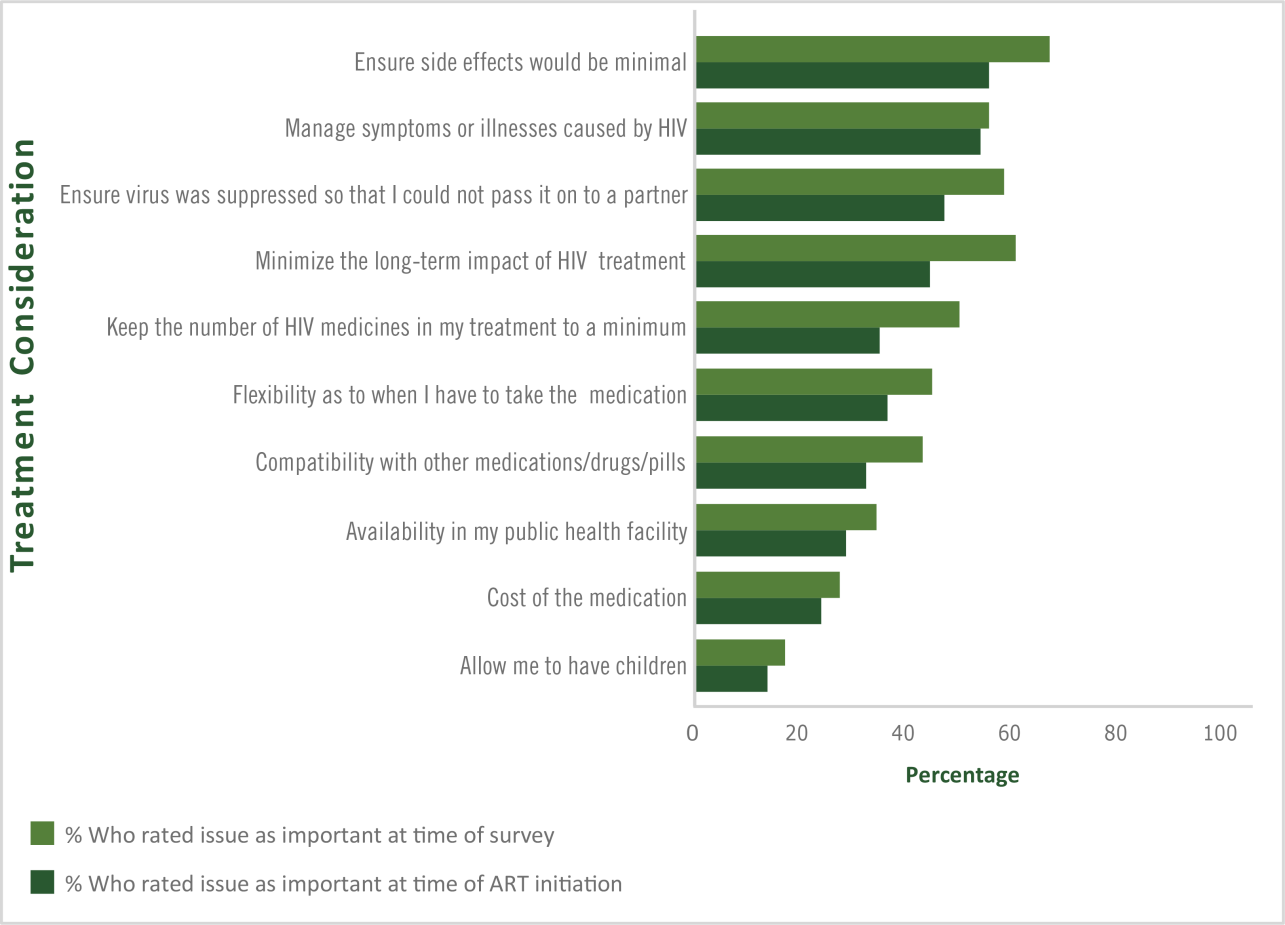
Perceived treatment priorities at initiation of antiretroviral therapy and at the time of the Positive Perspectives survey among treatment experienced people from 24 countries who had been living with HIV for 2 years or longer (n = 1,624), 2019.

**Polypharmacy and its associations with new concerns toward medications**. Among people who were 2 or more years post HIV/AIDS diagnosis (n = 1,624), a comparison of treatment priorities at time of initiating ART versus priorities at the time of our survey revealed that the 2 concerns with the largest absolute increase in perceived importance were minimizing the long-term impact of HIV treatment (16.1 percentage points difference) and reducing the number of medications in ART (15.0 percentage points difference) (Figure 2). Prevalence of new ART-related concerns among people who were 2 or more years post HIV/AIDS diagnosis and who did not report those concerns at ART initiation was as follows: risk of side effects (46.6%, 337 of 724); risk of long-term effects, including potential damage to organs such as kidneys or bones (45.5%, 411 of 904); risk of transmission to a partner (40.4%, 347 of 860); perceived need to reduce number of medications in ART (35.7%, 378 of 1,059); risk of drug–drug interactions (29.4%, 323 of 1,100); management of HIV-related illnesses (29.4%, 221 of 751); flexibility with meals or time taken (29.0%, 300 of 1,035); availability/accessibility (19.5%, 226 of 1,162); cost (13.9%, 172 of 1,239); and childbearing (10.2%, 143 of 1,401). Polypharmacy and current experience of side effects were significantly associated with new concerns regarding drug–drug interactions, and side effects (Table 4). Within adjusted analyses, PLHIV reporting polypharmacy had significantly increased odds of having new concerns about the risk of drug–drug interactions (AOR = 1.32; 95% CI, 1.02–1.71), and of side effects (AOR = 1.31, 95% CI, 1.02–1.68). PLHIV who perceived themselves as overmedicated also had higher odds of reporting new concerns about the following: number of medications in their ART and the need to keep them at a minimum (AOR = 1.54; 95% CI, 1.22–1.95), risk of drug–drug interactions (AOR = 1.38; 95% CI, 1.08–1.77), flexibility of dosing (AOR = 1.41; 95% CI, 1.10–1.82), and availability of ART (AOR = 1.39; 95% CI, 1.05–1.85). PLHIV who were experiencing side effects from their ART at the time of our survey had higher odds of reporting new concerns regarding risk of side effects (AOR = 1.53; 95% CI, 1.20–1.96) and concerns about viral suppression and disease transmission to a partner (AOR = 1.29; 95% CI, 1.01–1.65).

**Table:** 

Characteristic	Rated Issue Important at Time of ART Initiation, %	Rated Issue Important at Time of Survey, %
Allow me to have children	13.7	17.0
Cost of the medication	23.7	27.2
Availability in my public health facility	28.5	34.2
Compatibility with other medications/drugs/pills	32.3	42.9
Flexibility as to when I have to take the medication	36.3	44.7
Keep the number of HIV medicines in my treatment to a minimum	34.8	49.8
Minimize the long-term impact of HIV treatment	44.3	60.4
Ensure virus was suppressed so that I could not pass it on to a partner	47.0	58.2
Manage symptoms or illnesses caused by HIV	53.8	55.4
Ensure side effects would be minimal	55.4	66.8

**Table 4:** Experience of Side Effects, Polypharmacy^a^, Perceived Overmedication, and Cut-Down Attempts as Predictors of New Treatment Concerns^b^ Among People Living With HIV for 2 Years or Longer (N = 1,624), Positive Perspectives Study, 2019^c^

New Treatment Concern	Report of Experiencing Side Effects From ART, AOR (95% CI)	Polypharmacy, AOR (95% CI)	Perceived Overmedication, AOR (95% CI)^d^	Cut-Down Attempts, AOR (95% CI)
Keep number of HIV medicines in ART to a minimum	1.10 (0.86–1.39)	1.26 (0.99–1.61)	1.54 (1.22–1.95)^e^	0.92 (0.72–1.18)
Minimize the long-term impact of HIV treatment	1.06 (0.84–1.33)	1.15 (0.91–1.45)	1.12 (0.89–1.40)	0.82 (0.64–1.04)
Compatibility with other medications	1.09 (0.84–1.40)	1.32 (1.02–1.71)^e^	1.38 (1.08–1.77)	0.84 (0.65–1.10)
Allow me to have children	1.31 (0.92–1.86)	1.03 (0.71–1.49)	1.38 (0.98–1.96)	1.69 (1.16–2.44)^e^
Viral suppression to prevent transmission to partner	1.29 (1.01–1.65)^e^	1.27 (0.99–1.63)	1.21 (0.95–1.54)	0.90 (0.70–1.16)
Flexibility (eg, time of day, with or without food)	1.08 (0.83–1.40)	1.40 (1.07–1.83)^e^	1.41 (1.10–1.82)	0.87 (0.67–1.15)
Minimal side effects	1.53 (1.20–1.96)^e^	1.31 (1.02–1.68)^e^	1.14 (0.90–1.46)	0.81 (0.62–1.04)
Availability of ART in my public health facility	1.14 (0.85–1.52)	1.34 (0.99–1.79)	1.39 (1.05–1.85)	1.18 (0.87–1.59)
Medication cost	0.91 (0.66–1.27)	1.34 (0.96–1.86)	1.09 (0.79–1.50)	1.24 (0.89–1.73)
Manage HIV symptoms or illnesses	1.01 (0.76–1.36)	1.22 (0.90–1.64)	1.17 (0.88–1.56)	1.14 (0.84–1.54)

Abbreviations: AOR, adjusted odds ratio; ART, antiretroviral therapy; CI, confidence interval.

a Polypharmacy was defined as taking 5 or more pills per day for HIV or non-HIV conditions, or currently taking medicines for 5 or more conditions, including HIV.

b A new treatment concern was one that was not deemed important by the survey respondent at ART initiation but was considered a priority at the time of this study.

c Among treatment experienced people living with HIV. Separate logistic regression models were fitted for the different outcomes, controlling for age, geographic region, urbanicity, education, and sexual orientation.

d To assess perceived overmedication (cognitive proxy), we used a set of 6 questions that assessed respondents’ anxieties regarding their current HIV medications: 1) short-, medium-, and long-term effects of including worries about long-term side effects; 2) having to take more and more medications; 3) potential interactions with other medications; 4) effect on body and/or body shape; 5) effect on overall health and well-being; 6) unknown long-term effects. Being worried on at least 5 of these issues was subjectively classified as the respondent’s acute awareness of the number of medications they were taking. To assess cut-down attempt (behavioral proxy) we classified people as having taken deliberate efforts to reduce the number and effects of their HIV medications if they skipped taking their medications in the past 30 days because they were concerned about short- or long-term side effects or if they completely switched medications to reduce the number taken.

e Significant at *P* < .05.

**Determinants of openness to taking ART with fewer medicines.** Most (75.1%) PLHIV were aware of the number of medicines their ART medications contained (Table 2). Of all participants, 73.1% were open to switching to an ART that contained fewer medications as long as their viral load was suppressed. Willingness to switch was highest among men who have sex with men (77.9%, 742 of 953) vs men who have sex with women (63.8%, 268 of 420) and women who have sex with men (63.8%, 263 of 369) (*P* < .001); people aged 50 or older (79.9%, 518 of 648) compared with those younger than 50 (70.1%, 1,026 of 1,464) (*P* < .001); those living with HIV the longest (ie, diagnosed pre-2010 [76.4%] compared with those diagnosed between 2010 and 2016 [70.1%, 564 of 805] or 2017 and 2019 [72.5%, 354 of 488]) (*P* = .014), and those living in metropolitan areas (76.3%, 893 of 1,170) compared with nonmetropolitan areas (69.1%, 651 of 942) (*P* < .001). Furthermore, willingness to switch increased with increasing number of comorbidities and was 68.8%, 73.3%, and 77.9% among those reporting 0, 1, or 2 or more comorbidities, respectively (*P* < .001). Interest in taking fewer medicines increased with increasing number of concomitant medications; 70.3% (850 of 1,209) of people on ART only and no other medication indicated willingness to switch to ART with fewer medicines. This percentage increased to 77.8 (326 of 419) among those with 1 medication in addition to ART and to 76.0% (368 of 484) among those with 2 or more other medications.

Overall, 73.4% (n = 1,550) of study participants had ever switched ART since starting treatment. Top reasons for switching among those who had ever switched were to reduce the severity or frequency of side effects (45.3%) and to reduce the number of pills (35.0%) or number of medicines (26.8%) taken. Other specified reasons were because of ineffectiveness or resistance (24.4%), drug–drug interactions (18.6%), or cost (14.8%). Of all participants, 32.0% reported skipping their ART on at least one occasion in the past 30 days out of concerns for immediate or long-term side effects.

In total, 44.3% of participants experienced side effects from their ART, and 41.8% of all participants felt HIV had a negative effect on their life. Specific ART-related concerns among the overall sample included risks of long-term effects of HIV medicines (66.6%) and the risk for drug–drug interactions (48.5%) (Table 2). 

## Discussion

Polypharmacy was strongly associated with poorer health-related quality of life outcomes among PLHIV, independent of the presence of existing comorbidities. These findings were consistent both among those reporting and not reporting virologic suppression, although prevalence estimates of poor health outcomes were significantly higher among the latter. The finding of significant associations even among those reporting viral suppression supports the idea espoused in the fourth 90 of the UNAID’s 90–90-90 framework that quality of life is not about just virologic control. In our study, one of the most common reasons PLHIV switched ART in the past was to reduce the number of medicines or pills they had to take, and 2 in 3 PLHIV were willing to switch to an ART with fewer medications as long as their viral load was suppressed. Concurrent use of multiple medications may considerably increase risk of drug–drug interactions, especially among people with concomitant medications (22–24), and this concern was well reflected in our study. Recent studies have established that the number of drug–drug interactions increases with the number of medications (22–24). Most non-HIV conditions reported by PLHIV in our study were chronic conditions largely found in the general population, such as mental health conditions, hypertension, and hypercholesterolemia, which may require long-term management with medications. But even besides concerns regarding the risk of drug–drug interactions, increased use of medicines was associated with poor treatment satisfaction among PLHIV in our study.

Our findings emphasize that treatment needs are constantly evolving; continuous communication between patients and providers is therefore critical. For example, PLHIV experiencing side effects from their ART at the time of the study had significantly higher odds of reporting worries about the risk of side effects as a new concern relative to when they started ART. The most common new concerns among PLHIV were the risks of side effects, long-term effects, disease transmission to a partner, and the number of medicines in ART. Number of medicines in ART, along with concerns about long-term effects of HIV medicines, had the largest absolute increase as important treatment considerations at the time of our survey relative to time of ART initiation among treatment experienced PLHIV. These evolutions in patient preferences with age or onset of other comorbidities highlight the need to constantly re-evaluate current and future treatment needs of PLHIV on the basis of patient-specific risks of comorbidities, co-existing conditions, concomitant medications, and patient concerns about treatment. A proactive treatment plan that considers the totality of a patient’s current treatments and risks can result in a more holistic care that optimizes patient well-being.

Health-related quality of life outcomes are harder to quantify as measures of success, and most clinicians have limited power over upstream factors that affect overall well-being, such as socio-economic status. However, our study demonstrates the role of drug treatment on well-being within the context of increased medicines and how providers can positively affect care. By assessing patients holistically, considering their treatment needs and concerns, and streamlining treatments where appropriate, clinicians may contribute to improving patients’ health-related quality of life. Alleviating anxiety through regular and continued communication and education may also reduce the worries and concerns reported in this study. 

Our study suggests that patients were willing to switch to treatments with fewer medications, despite more than 2 in 3 reporting treatment satisfaction. Regardless of a patient’s current viral load, constant review and assessment of treatment plans may be beneficial because patient needs and understanding of their medication evolve over time. Health care providers should be encouraged to simplify treatments where possible, discuss patients’ treatment concerns, and share decision making with them, as well as provide them with information on newer treatments that may be beneficial to them. 

HIV specialist clinicians can work holistically as part of a multidisciplinary team to address the various treatment-related concerns, worries, and anxieties identified in our study, especially among patients at greatest risk of receiving fragmented care, including older people, those with multiple disorders, those living in poverty, the uninsured, and patients in rural areas. The metrics for evaluating successful disease management, should focus not only on clinical endpoints, but also on patient-oriented outcomes and treatment satisfaction to ensure overall wellbeing. Enhanced and sustained efforts in this regard will ensure progress toward the National HIV/AIDS strategy, a 5-year plan developed by the US department of Health and Human Services in response to the HIV epidemic (19).

A strength of our study is its use of a standardized protocol in 24 countries. To the best of our knowledge, this is one of the largest studies of PLHIV to assess patient-centered outcomes, including aspirations and attitudes toward treatment. Nonetheless, our study had limitations. First, the cross-sectional design limited any causal inferences. Second, self-reported measures such as viral load may have resulted in misclassification. For example, we classified both people reporting their viral load as detectable or unsuppressed and those who reported they did not know or who refused to answer as virally failing, which may result in misclassification of those with indeterminate status; however, the extent of bias is likely small because of the small percentage this group comprised. Third, the sample may be biased because of nonprobabilistic sampling and the use of a web-based questionnaire. Finally, only a limited number of countries were included. For example, although Africa bears the largest HIV burden in the world, no country in sub-Saharan Africa was included in our analyses.

Approximately 2 in 5 PLHIV surveyed reported polypharmacy, which was significantly associated with poorer health-related quality of life outcomes. A high number of study participants also reported being worried about taking more medicines as they grew older; many were open to switching to ART with fewer medicines, especially people with kidney and bone disorders. Clinicians should consider addressing patient concerns and worries along with treatment. Doing so may help improve health-related quality of life among PLHIV and accelerate progress toward meeting UNAID’s fourth 90.
